# Tracking Modified Vaccinia Virus Ankara in the Chicken Embryo: In Vivo Tropism and Pathogenesis of Egg Infections

**DOI:** 10.3390/v10090452

**Published:** 2018-08-24

**Authors:** Martin C. Langenmayer, Anna-Theresa Lülf-Averhoff, Silvia Adam-Neumair, Gerd Sutter, Asisa Volz

**Affiliations:** 1 Institute for Infectious Diseases and Zoonoses, LMU Munich, 80539 Munich, Germany; martin.langenmayer@lmu.de (M.C.L.); anna.luelf@campus.lmu.de (A.-T.L.-A.); adam-neumair@micro.vetmed.uni-muenchen.de (S.A.-N.); asisa.volz@micro.vetmed.uni-muenchen.de (A.V.); 2 German Center for Infection Research (DZIF), Partner Site Munich, 80539 Munich, Germany

**Keywords:** biodistribution, CAM, chorioallantoic membrane, host range, immunohistochemistry, poultry, poxvirus, virus propagation

## Abstract

The Modified Vaccinia virus Ankara (MVA) is a highly attenuated vaccinia virus serving as a promising vector vaccine platform to develop vaccines against infectious diseases. In contrast to the well-established replication deficiency and safety of MVA in mammals, much less is known about MVA infection in avian hosts. Here, we used a recombinant MVA expressing fluorescent reporter proteins under transcriptional control of specific viral early and late promoters to study in vivo tropism, distribution, and pathogenesis of MVA infections in embryonated chicken eggs. The chorioallantoic membrane (CAM) of embryonated chicken eggs was inoculated with recombinant MVA, MVA or phosphate-buffered saline. The infection was analyzed by fluorescence microscopy, histology, immunohistochemistry, and virus titration of embryonic tissues. After infection of the CAM, MVA spread to internal and external embryonic tissues with the liver as a major target organ. Macrophages and hematopoietic cells were identified as primary target cells of MVA infection and may be involved in virus spread. Increasing doses of MVA did not result in increased lesion severity or embryonic death. Despite MVA generalization to embryonic tissues, the CAM seems to be the major site of MVA replication. The absence of considerable organ lesions and MVA-associated mortality highlights an excellent safety profile of MVA in chicken hosts.

## 1. Introduction

Modified Vaccinia virus Ankara (MVA) was originally generated by Anton Mayr and Eberhard Munz in the 1960s [1]. The starting point was a study encompassing serial infection experiments in cultures of primary chicken embryo fibroblasts (CEF) with a standard vaccinia virus (VACV) strain, the Chorioallantoic VACV Ankara (CVA), being used for vaccination against smallpox in Turkey and Germany. After over 516 passages in CEF, a highly attenuated laboratory virus had emerged which was renamed MVA. The new virus differed from the parental CVA in several biomarkers of infection including the tropism in cell culture, the behavior in embryonated chicken eggs and the loss of virulence for mice, and rabbits [2]. The attenuated phenotype of MVA infection is associated with substantial changes in the viral genome involving large genomic deletions and fragmentation, truncation and deletion of multiple open reading frames typically found in VACV genomes [3,4]. In consequence, MVA is replication-deficient in mammalian hosts and productively grows in only a handful of cell cultures [4,5,6,7,8,9].

Since the 1990s, MVA has been investigated as a safe vector for the development of recombinant viral vaccines against infectious diseases and cancer. Moreover, MVA is promoted as modern and safe vaccine against smallpox to replace the earlier and fully replication competent VACV vaccines (for review, see [10]).

In contrast to the well-established replication deficiency and safety of MVA-based vector vaccines in mammals, much less is known about MVA infection in avian hosts. Only few studies have been conducted highlighting the usefulness of recombinant MVA as poultry vaccine against various infectious diseases. A single intramuscular inoculation of a recombinant MVA vaccine delivering the hemagglutinin antigen of highly pathogenic avian influenza virus A/Vietnam/1194/04 can protect chickens from a lethal challenge with a high dose of highly pathogenic A/duck/Vietnam/TG24-01/2005 [11]. Vaccination with an ancestral hemagglutinin H9 antigen delivered by recombinant MVA was evaluated against low pathogenic avian influenza virus subtype H9N2 infection in the chicken model [12]. Furthermore, an MVA vector vaccine expressing the capsid protein VP2 of infectious bursal disease virus is tested as a candidate vaccine in chicken [13,14]. Further experimental studies attempted in ovo vaccinations using an adenovirus and recombinant MVA expressing avian influenza T cell antigens. Upon vaccine regime optimization, MVA vector vaccine was inoculated into the amniotic cavity of the embryonated chicken egg and no adverse effects were observed [15]. Overall, however, there is still very little experience with regard to MVA infection and MVA distribution in chickens.

Although MVA has been adapted to grow in chicken cells and MVA vaccines have been amplified in embryonated eggs [16], MVA infection of the chicken embryo has not been studied in detail using modern methodologies.

Here we studied the in vivo tropism, distribution, and pathogenesis of MVA infections in embryonated chicken eggs. We took advantage of an MVA vector expressing two fluorescent reporter proteins and used whole organ imaging, virus titration, histology and immunohistochemistry to monitor the outcome of infections with various amounts of MVA. We show that MVA infection of chicken embryos leads to a generalized spread of virus and to the infection of several types of cells, including macrophages, hepatocytes and cells of the hematopoietic tissues. Surprisingly, MVA infection seems to be well tolerated because even high dose inoculations did not lead to increased incidence of embryonic death.

## 2. Materials and Methods

### 2.1. Viruses

To follow MVA distribution in the chicken embryo, a previously characterized recombinant MVA-GFP-mCherry was used as a reporter virus [17]. Briefly, this recombinant virus was generated using standard methods and a clonal isolate (F6) of MVA as the backbone virus [18]. MVA-GFP-mCherry expresses synthetic gene sequences encoding a green fluorescent protein (GFP) under transcriptional control of the vaccinia virus (VACV)-specific early promoter Pvgf and a red fluorescent protein (mCherry) regulated by the VACV-specific late promoter P11. The MVA clonal virus isolate F6 (MVA) served as non-recombinant infection control. Phosphate-buffered saline (PBS) served as a negative control.

### 2.2. Egg Preparation and CAM Infection

Fertilized eggs from a specific-pathogen-free (SPF) chicken flock (Valo Biomedia, Osterholz-Scharmbeck, Germany) were stored at 16 °C for two days prior to incubation. Eggs were incubated at 37.9 °C and 45% humidity in an incubator (MP GTFS, Grumbach, Schwabach) with automated turning of eggs. Eggs were inoculated at embryonic day 11 (ed11) of incubation. Shells were wiped with ethanol and perforated over the air chamber with a power tool. At the inoculation site, a small triangle of the shell was removed. Using a sterile fine dosage syringe with a 30G needle, the shell membrane was perforated and 100 µL of inoculum deposited between the CAM and the inner shell membrane. The hole in the shell was sealed with a transparent tape (Tesafilm, tesa SE, Norderstedt, Germany) and the eggs further incubated without automated rotation to promote sterility at the inoculation site.

Eggs were CAM-inoculated with 10³ infectious units (IU) MVA or MVA-GFP-mCherry per embryo. Control embryos were mock-infected with PBS. On days 2–7 post inoculation (2–7 dpi), embryos were necropsied. The experiments were carried out in accordance with institutional and national guidelines for work with genetically modified organisms. No ethical approval was required for this study.

### 2.3. Necropsy and Imaging

At necropsy, the following organs were imaged: Brain, CAM, gonads, heart, intestines, kidneys, liver, lung, proventriculus, spleen, ventriculus, and yolk sack. Proliferative nodules (pocks) on the CAM were scored according to their size: small: ≤1 mm; moderate: 2–5 mm; large: >6 mm.

Organs of MVA-GFP-mCherry infected embryos on petri dishes were examined with a ChemiDoc™ MP Imaging system (Bio-Rad Laboratories) and the MacroIllumination Imaging System (Lightools, Encinitas, CA, USA).

Images of native tissue preparations, histological and immunohistochemical slides were taken with an inverted fluorescence microscope (Keyence BZ-X700, Keyence, Neu-Isenburg, Germany) with GFP filter (excitation wavelength 470/40 nm, absorption wavelength 525/50 nm), TexasRed filter (excitation wavelength 560/40 nm, absorption wavelength 630/75 nm), and DAPI filter (excitation wavelength 360/40 nm, absorption wavelength 460/50 nm).

After imaging, CAM and organs were processed for histologic examination and viral titration.

### 2.4. Histology and Immunohistochemistry

Tissues for histology were routinely paraformaldehyde-fixed, paraffin-embedded, cut and sections stained with hemalum-eosin.

To keep the tension of the CAM during fixation and embedding process, CAMs were dissected with a modified version of Voss’ technique [19]. The fenestration of the eggshell was enlarged and CAMs were clamped between two metal washers taped to a forceps. CAMs were extracted, the two washers disconnected from the forceps and the CAMs including the washers were fitted into an embedding cassette and fixed with a clip. CAMs were trimmed after embedding. CAMs for Kul01-immunhistochemistry (IHC) were fixed in zinc-salt fixative [20].

IHC was performed with preceding avidin-biotin-block and without epitope retrieval [21]. For macrophage detection, a mouse monoclonal antibody was used (1:100, Kul01, Southern Biotech, 8420-01, Birmingham, AL, USA). For MVA detection, a polyclonal rabbit anti-vaccinia antiserum was used (1:1000, Acris, BP1076, Herford, Germany). Biotinylated secondary antibodies were a goat-anti-mouse Fab-fragment (1:200, Jackson Immunoresearch, 115-067-003, Cambridge, UK) and a goat anti-rabbit antibody (1:200, Vector, BA-1000, Burlingame, CA, USA). Peroxidase-complexed avidin-biotin (ABC-HRP, Vector, PK-6100) and diaminobenzidine were used as visualization and hemalum as counterstain. MVA-Kul01-double-IHC was done sequentially, first MVA-IHC with Streptavidin Alexa Fluor 568 (1:100, ThermoFisher, S-11226, Waltham, MA, USA), followed by avidin-biotin-block and Kul01-IHC with ABC-HRP and tyramide signal amplification with Alexa Fluor 488 (ThermoFisher, B40953) and nuclear DAPI-staining. Mouse IgG (Vector, I-2000) and rabbit-anti-*Escherichia coli* (B0357, Dako, Hamburg, Germany) were used as negative controls. Tissues containing macrophages (Kul01-IHC: spleen, bursa) or orthopoxviruses (MVA-IHC) served as positive controls.

### 2.5. Virus Titration

Organs of chicken embryos were weighed, freeze-thawed three times and homogenized with PBS in a microtube for 45 s at lowest level (Retsch TissueLyser MM 300; Qiagen GmbH, Hilden, Germany). After centrifugation (1 min; 1500 rpm; 4 °C), supernatants were stored at −80 °C.

Virus infectivities were determined via routine plaque assays performed in duplicate [18]. In 6-well-plates, confluent CEF monolayers were infected with serial 10-fold dilutions of the organ supernatants. After two hours at 37 °C, the cells were washed with PBS. After washing, cells were incubated at 37 °C for two days with virus growth medium [18]. The infected cells were fixed with acetone-methanol and incubated with polyclonal rabbit anti-vaccinia antiserum (1:1000, Acris, BP1076), followed by peroxidase-conjugated goat anti-rabbit antibody (1:5000, Jackson ImmunoResearch, 111-035-035). Infectious foci were visualized with TrueBlue, counted, calculated, log10-transformed, and indicated in IU/g organ.

### 2.6. Serum Analysis

For serum analysis, embryonated eggs were CAM inoculated with 10^9^ IU MVA or MVA-GFP-mCherry and blood samples were collected by piercing the chorioallantoic veins at 4 dpi with a 30G needle connected to a fine dosage syringe. Serum samples were stored at −80 °C. Sera were diluted with saline to obtain a minimal amount of 100 µL and enzyme activities were calculated for the original concentration. Six of eleven undiluted sera were analyzed in parallel to control dilution. Aspartate transaminase (AST), glutamate dehydrogenase (GLDH), and lactate dehydrogenase (LDH) activities were analyzed with a Cobas Integra 400 plus (Roche, Grenzach-Wyhlen, Germany) at the laboratory of the Clinic of Small Animal Medicine, Centre for Clinical Veterinary Medicine, LMU Munich.

### 2.7. Data Analysis

Statistical tests and calculations were performed with Microsoft Excel (Microsoft Office 2016, Redmond, WA, USA) and GraphPad Prism version 5.04 for Windows (GraphPad Software, La Jolla, CA, USA).

## 3. Results

### 3.1. MVA Infection of the Chorioallantoic Membrane (CAM) in Chicken Eggs

Following established methodology for CAM infections with poxviruses [22], we inoculated 10³ IU MVA or MVA-GFP-mCherry and started with the characterization of the infection site.

**Figure 1:**
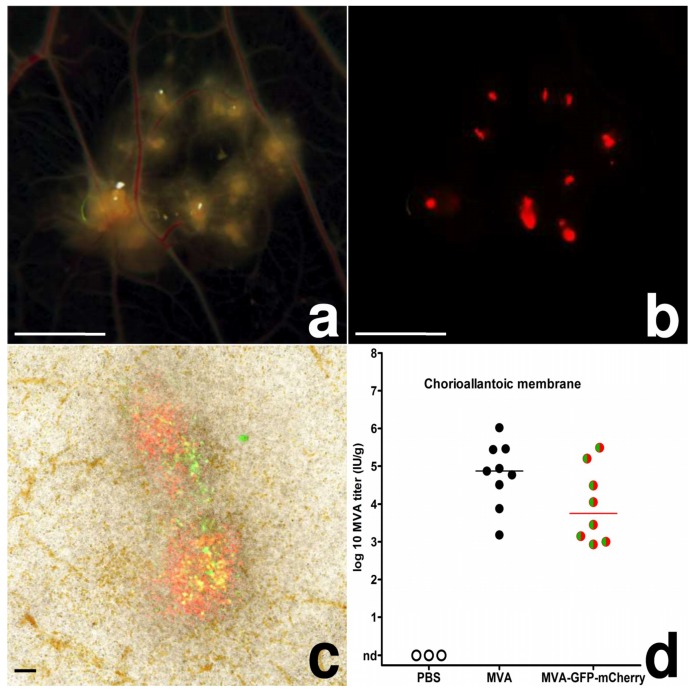
MVA-infection, chicken chorioallantoic membrane; (**a**) CAM, 4 dpi, multiple pocks along the vascular tree, bar = 5 mm; (**b**) CAM, 4 dpi, red fluorescence of the same pocks, bar = 5 mm; (**c**) CAM, 3 dpi, native preparation on a slide, green and red fluorescence of two pocks, bar = 100 µm; (**d**) scatter dot plot of MVA titers in the CAM after infection with 10³ IU. The lines represent medians, nd = not detected.

As early as 2 dpi, MVA inoculated CAMs displayed defined areas of multiple white, proliferative nodules with diameters of ~1.0–3.5 mm (pock lesions; Figure 1a). In MVA-GFP-mCherry inoculated CAMs, we found these pock lesions to be consistently associated with the detection of green and strong red fluorescence (Figure 1b,c). These lesions were clearly orientated along the vascular trees, and in some of the embryonated eggs, we observed the development of secondary pocks on the CAM distant from the primary site of infection. The detection of green and red fluorescence in CAM tissues indicated the production of the fluorescent reporter proteins and thus the unimpaired expression of early and late classes of viral genes. In addition, the finding of substantial virus loads in CAMs infected with MVA or MVA-GFP-mCherry confirmed the productive replication of the viruses (Figure 1d).

**Figure 2:**
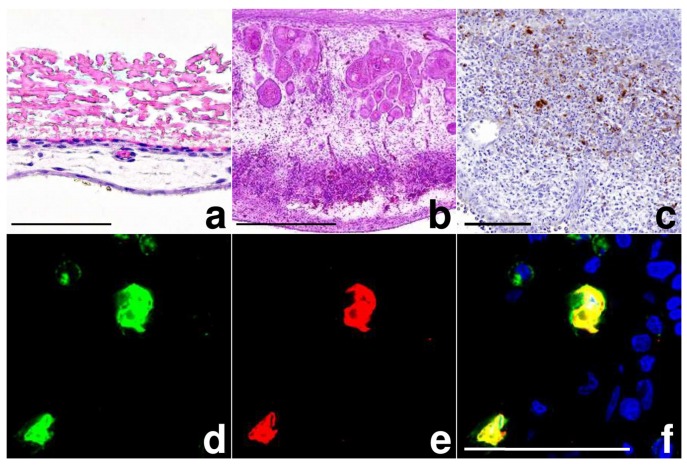
Histology of MVA-infection, chicken chorioallantoic membrane; (**a**) CAM, normal, from top: thick inner shell membrane, single layer of ectoderm with interwoven capillaries, mesoderm with vessel, single layer of entoderm, bar = 50 µm; (**b**) CAM, 7 dpi, pock lesion with massive thickening of ectoderm and mesoderm, bar = 500 µm; (**c**) CAM, 2 dpi, MVA-immunohistochemistry (IHC), thickening of mesoderm with inflammation and MVA antigen (brown) in mesoderm, bar = 100 µm; (**d**–**f**) CAM, 4 dpi, Kul01-vaccinia virus-double-IHC, macrophages (Kul01+; green) containing MVA antigen (red), in the overlay displayed yellow, nuclear staining with DAPI, bar = 50 µm.

Histological and immunohistochemical examination revealed considerable changes in the MVA infected CAM tissues including proliferation of resident cells (see below) and the presence of large numbers of inflammatory cells mainly consisting of macrophage-like cells and granulocytes. The proliferation of resident cells and the massive cellular influx at the site of the pock lesion increased the thickness of the CAM about 40-fold to ~1100 µm, compared to the ~25 µm thickness of normal CAM (Figure 2a,b). Moreover, due to the expansion of the pock lesions, the CAM partially separated from the shell membrane. The normally double-layered ectoderm was hyperplastic and contained many transmigrating macrophages and granulocytes targeting the inoculum between the ectoderm and the shell membrane. Later in infection, the ectoderm displayed cellular hypertrophy, pronounced hyperplasia, flattening of superficial cells and detachment from the basal layer. In large pocks, multiple newly-formed epithelial islands were found in the mesoderm. The mesoderm was considerably thickened, edematous and infiltrated by moderate to large numbers of granulocytes and macrophages. We detected multifocal angiogenesis and dilated mature vessels displaying intravascular leukocyte margination. Even early in infection, we found considerable proliferation of fibroblasts in the mesoderm leading to deposition of collagen fibers in late infection. Overall, the entodermal reactions to infection were less pronounced and consisted of only mild hyperplasia.

**Figure 3:**
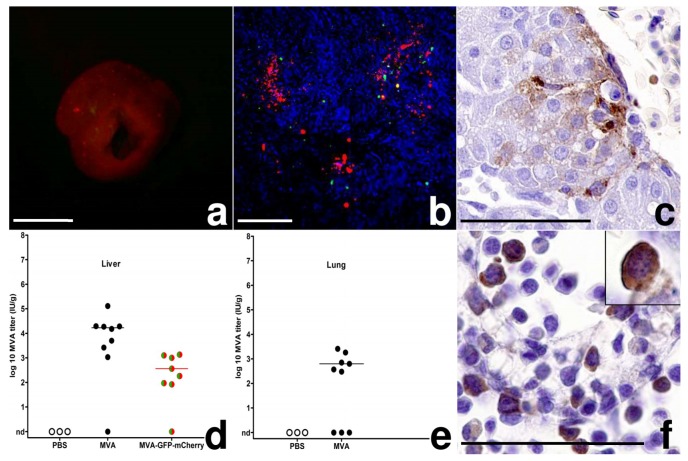
MVA-infection, chicken embryo; (**a**) Liver, 3 dpi, multifocal random red-fluorescent spots, bar = 5 mm; (**b**) Liver, 3 dpi, native preparation on a slide, green and red fluorescence of foci, hepatocyte autofluorescence in blue, bar = 100 µm; (**c**) Liver, 2 dpi, MVA antigen in hepatocytes, MVA-IHC, bar = 50 µm; (**d**,**e**) Scatter dot plot of MVA titers in the liver and lung after infection with 10³ IU. The lines represent medians, nd = not detected (**f**) Bone marrow, 6 dpi, intra- and extravascular hematopoietic cells with MVA antigen, inset: 2 dpi, circulating hematopoietic cell in the liver, MVA-IHC, bar = 50 µm.

Using immunohistochemistry, MVA antigen was detected at 2 dpi in the cytoplasm of ectodermal and mesodermal cells including many putative macrophages and to a lesser extent fibroblasts (Figure 2c). We also detected mCherry antigen in the same cells, corroborating the results of the MVA-IHC and the fluorescence experiments. Positive staining using Kul01-MVA-double-IHC suggested that the majority of MVA antigen-positive cells were macrophages (Figure 3a–c) confirming Kul01+ macrophages as main target cells for MVA infection in the CAM.

### 3.2. MVA Spread to the Chicken Embryo and the Extraembryonic Tissues

To assess MVA spread from the CAM as the primary site of infection, we analyzed the embryonic organs and extraembryonic tissues for signs of viral replication using the reporter virus MVA-GFP-mCherry. On day 3 pi, we observed multifocal miliary red-fluorescent spots randomly distributed in ~10% of the livers. These red-fluorescent foci also contained fewer cells displaying green fluorescence as revealed upon microscopic examination (Figure 3a,b). Using histology and immunohistochemistry, we clearly detected MVA infected hepatocytes as evidenced by virus antigen being multifocally and randomly distributed within the cytoplasm of the infected cells (Figure 3c). Moreover, some virus-infected hepatocytes demonstrated typical signs of necrosis and were clearly associated with macrophage-like cells and/or granulocytes. Most MVA antigen-positive cells, however, were without signs of inflammation or detectable cellular injury. We also detected mCherry antigen in hepatocytes, corroborating the expression of late viral genes and the results of the MVA-specific IHC and the imaging experiments. The finding of MVA infected liver cells supported the generalized spread of MVA from the inoculation site to the liver. To further confirm a generalized MVA infection, we analyzed viral loads in livers and lungs and found 10^2^ to 10^4^ IU/g tissue MVA or MVA-GFP-mCherry in livers and lungs of most infected embryos (Figure 3d,e). Interestingly, when we correlated log10-transformed virus titers from CAM and liver, we identified a significant moderate to strong positive correlation (Pearson-correlation; 10^3^ IU: *r* = 0.75, *p* = 0.001). The correlation of high MVA titers in the CAM with high MVA titers in the liver suggested a direct connection between the infections of these two embryonic tissues.

However, we failed to detect red fluorescence when imaging other organs of the chicken embryos. To characterize the putative spread of MVA infection in more detail, we assessed a large set of organ samples from infected embryos by histology and immunohistochemistry. Indeed, already at 2 dpi we found cell-associated MVA antigen in the tissues of bursa fabricii, heart, intestines, kidneys, skeletal muscle, skin, and ventriculus in many different cell types including endothelial cells, fibroblasts, myocytes (cardio-, leio-, rhabdo-), and glomerular mesangial cells. Moreover, we detected MVA antigen in hematopoietic cells in the yolk sack and the bone marrow. Both extravascular (granulopoiesis) and intravascular (erythropoiesis) hematopoietic cells displayed cytoplasmic MVA antigen (Figure 3f). In the bone marrow and yolk sack, we detected few overall cytopathic effects with rare detection of karyorrhexis and single cell necrosis. Additionally, we found single intravascular hematopoietic precursor cells containing cytoplasmic MVA antigen suggesting vascular distribution of the virus through different organs, already at early points of infection (2 dpi, Figure 3f, inset). Furthermore, mainly in liver and lung, the perivascular foci of heterophilic granulopoiesis were mildly to moderately expanded, probably as a result of an increased inflammation of the CAM. Mock-infected control embryos occasionally displayed mild hemorrhage or inflammation at the inoculation site, but were without lesions or MVA antigen in any tissues or organs.

**Figure 4:**
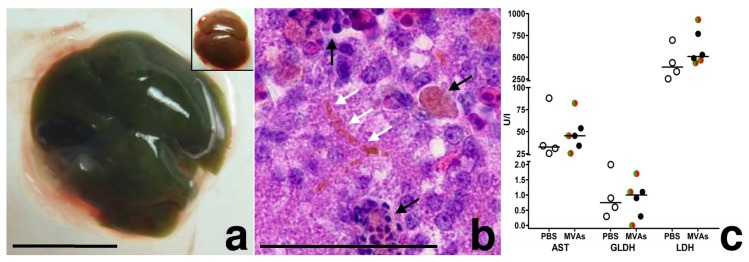
MVA infection, chicken embryo; (**a**) Liver, 4 dpi, green color, inset: Liver, 4 dpi, mock-infected embryo, bar = 5 mm; (**b**) Liver, 4 dpi, different stages of erythrophagocytosis (black arrows) and plugs in bile canaliculi (white arrows), bar = 50 µm; (**c**) Scatter dot plot of serum enzymes of infected (MVA: black circles; MVA-GFP-mCherry: green/red circles) and control chicken embryos (PBS: empty circles). The lines represent medians.

Interestingly, we noticed another macroscopic alteration in the infected livers. Starting at 5 dpi, livers of MVA-infected embryos (Figure 4a), and in some animals also kidneys and allantoic fluid, displayed a clearly visible green color. Other macroscopic overt signs of infection or gross lesions were not detected. In histologic sections, from 3 dpi on, we detected many Kupffer cells performing multifocal erythrophagocytosis. From 4 dpi on, consistently, Kupffer cells contained cytoplasmic bile pigment, and bile plugs were detected in canaliculi and small bile ducts (Figure 4b). These histological findings are consistent with the macroscopic observations in livers suggesting cholestasis in the liver and subsequent hyperbiliverdinemia as cause of the green color of the embryonic liver after MVA infection.

As we did not detect overt hepatocyte damage histologically, we further monitored for potential liver damage and comparatively analyzed activities of liver enzymes in four mock infected and six MVA infected embryos. As a positive control, we used an independently selected chicken embryo with macroscopically and histologically confirmed liver necroses. Aspartate transaminase (AST), glutamate dehydrogenase (GLDH), and lactate dehydrogenase (LDH) activities were within normal variation and displayed no major or significant differences (Mann Whitney test, *p* > 0.17–0.91) between PBS inoculated and MVA infected chicken embryos (Figure 4c). In contrast, the embryo with histologically confirmed liver necroses displayed a 10-fold increase of AST activity (465 U/L), 198-fold increase of GLDH activity (183 U/L), and 21-fold increase of LDH activity (9291 U/L) when compared to mean enzyme activities of PBS inoculated embryos.

### 3.3. Egg Infections Using Escalating MVA Doses

Since the data so far indicated a generalized spread of MVA after CAM inoculation, we assessed the effect of increasing doses of MVA or MVA-GFP-mCherry with regard to pathological findings and mortality in the chicken embryos. We infected embryonated eggs with 10^3^, 10^5^, or 10^7^ IU, candled the eggs twice daily, and analyzed the embryonated eggs after generalization of infection at the time point 4 dpi.

The infections with the three MVA doses induced the formation of pock lesions in the CAM with comparable sizes ranging in diameter between 2–5 mm. A significant increase in lesion size corresponding to escalating inoculation doses was not detected. Pearson’s correlation of log10-transformed viral titer in the CAM with pock lesion size was performed in the three dosing groups. There was no significant correlation (*r* = 0.15–0.41, *p* = 0.13–0.60). In addition, there were no detectable differences when comparing the lesion sizes induced by MVA or MVA-GFP-mCherry infections.

Upon pathological examination of the infected chicken embryos, we once again found no major change in gross lesions associated with the inoculation of different amounts of virus. Of note, until the day of analysis none of the 52 infected embryos had died from the infection. When monitoring the internal organs, the green color of livers seemed more intense in embryos inoculated with the higher doses of 10^5^ or 10^7^ IU MVA or MVA-GFP-mCherry. Analogously, we detected a higher number of foci of erythrophagocytosis and more accumulations of bile pigment in canaliculi and ducts in the liver sections from these animals.

**Figure 5:**
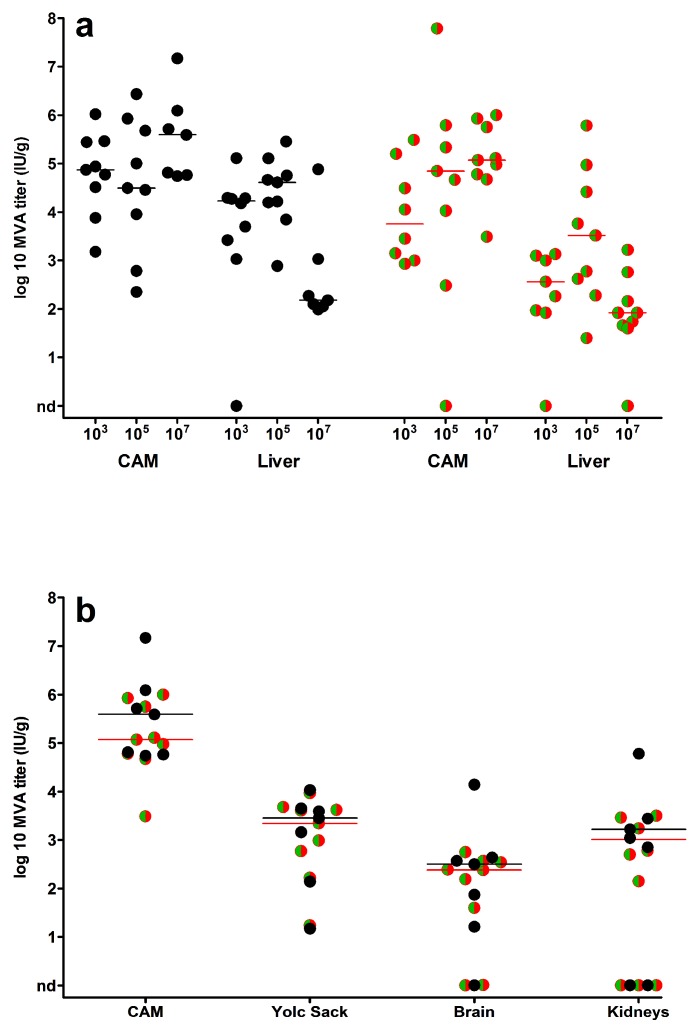
Virus loads in embryonic organs. Scatter dot plots of virus titers (IU/g tissue) in chicken embryos infected with MVA (black circles) or MVA-GFP-mCherry (green/red circles); (**a**) organ viral loads detected in CAMs and livers following inoculation with 10^3^–10^7^ IU virus or (**b**) in yolk sack, brains and kidneys after infection with 10⁷ IU virus. Infectious virus was not detected in organs of control embryos (PBS mock-infected). The lines represent the medians, nd = not detected.

Finally, we determined viral loads in CAMs, livers and in selected embryonic organs such as yolk sack, kidney and brain (Figure 5). Overall, the infections with MVA or MVA-GFP-mCherry resulted in very comparable amounts of infectious virus detectable in all organs. Overall, CAM and liver tissues contained the highest loads of infectious virus (Figure 5a). When we correlated log10-transformed viral loads in CAMs and livers in 10^5^ and 10^7^ IU inoculated embryonated eggs, there was a significant moderate positive correlation (Pearson correlation; 10^5^ IU: *r* = 0.52, *p* = 0.032; 10^7^ IU: *r* = 0.61, *p* = 0.005). Lower viral titers were found in yolk sack, kidney and brain (Figure 5b). Yet, the presence of readily detectable infectious virus in the majority of the samples indicated a generalized distribution of the viruses in the whole chicken embryo.

## 4. Discussion

In this study, we analyzed the pathogenicity and the in vivo tropism of MVA in chicken embryos. MVA displayed generalized spread from the primary site to the chicken embryo and the internal organs with a major focus on the infection of the embryonic liver. Interestingly, increasing infectious doses of MVA did not result in increased lesion severity or death of the chicken embryos. Different types of host cells were found permissive for infection and may support the spread of the virus within the chicken embryo.

MVA is a well-established third generation smallpox vaccine and serves as a promising viral vector for the development of candidate vaccines against infectious diseases or cancer [10]. After serial cell culture passages on CEFs, MVA was adapted to grow in avian cells and lost the ability to replicate productively in mammalian hosts [2,4]. However, there is still relatively little known about the pathogenesis and biodistribution of MVA in an avian host. This information, however, is important with regard to poultry vaccine development as well as to estimate a potential risk for the general environment in case of an unintentional release of genetically modified MVA vaccines [23]. Moreover, a more detailed understanding about the tropism of MVA in chicken might contribute to further elucidate poxvirus gene functions involved in the regulation of virus-host interactions.

Before the routine availability of cell culture methods, virologists often used infections of embryonated chicken eggs to amplify, titer, and characterize viruses. For poxviruses, the most common method was the CAM inoculation [24,25,26]. The histologic architecture of the CAM, three germinal layers consisting of ectoderm, mesoderm and entoderm, provides a broad range of different cell types as suitable viral targets. Thus, we used CAM inoculations to study the distribution, tissue tropism and cellular tropism of a recombinant MVA expressing green and red fluorescent proteins as specific markers for key steps in the VACV life cycle [17]. We confirmed unimpaired expression of MVA early and late genes upon infection of the CAM, when we detected green and red fluorescent cells in primary and secondary pock lesions. Productive MVA infection was further supported by immunohistochemistry and analysis of viral loads in the CAM. We detected ample amounts of viral antigen within the mesoderm of the CAM and an increase in viral load. Using the low infectious dose of 10^3^ IU, MVA seemed to multiply somewhat more efficiently in the CAM when compared to MVA-GFP-mCherry. As both viruses demonstrate identical growth properties in tissue culture [17], this observation may indicate that the expression of the two marker genes influences MVA-GFP-mCherry replication in an egg infection. Applying higher infectious doses for infection and monitoring viral load in other tissues we observed about equal titers of MVA and MVA-GFP-mCherry. Yet, the monitoring of replicative capacity needs further attention in other in vivo infection studies using MVA-GFP-mCherry.

With the use of a zinc-salt dependent fixative, which preserves fixation-sensitive epitopes [27,28], we demonstrated that MVA mainly targets Kul01+ macrophages [29] in chicken embryos. The preference of MVA for infection of human peripheral blood mononuclear cells (PBMC)-derived monocytes and dendritic cells is well-known from in vitro studies [30,31]. Recently, in vivo experiments also demonstrated the direct targeting of such antigen presenting cells in mice, ferrets and non-human primates [32]. When considering the potential of MVA vector vaccines for immunization of poultry or in ovo vaccination, the targeting of avian antigen presenting cells could also be beneficial to elicit an effective immune response.

The production of the fluorescent marker proteins allowed tracking of MVA within the embryo. At 3 dpi, green and red fluorescent foci in the liver initially indicated the infection of hepatic tissues and the generalized spread of MVA from the CAM as the primary site to the chicken embryo. Using immunohistochemistry, we identified hepatocytes as main MVA target cells in the liver and the titration of infectious virus in liver tissues suggested a productive MVA infection. The correlation of MVA titers in CAM and liver tissues further support the assumption that MVA is spreading from the CAM directly to the liver via the blood stream.

This is in line with our observation that primary pock lesions in the CAM are mostly located next to the vascular trees of the membrane. Developing from ed4 to ed12 the CAM becomes the major site of respiration in the avian embryo until ed19 [33]. Blood is oxygenated extraembryonically in the CAM and flows via the chorioallantoic vein into the embryo through the liver into the heart [34]. Our finding of MVA infection in hematopoietic cells further bolstered the hypothesis that MVA is distributed via the blood stream, because these cells are the progenitors of all blood cells. Furthermore, during development, these hematopoietic cells move between different compartments in the chicken embryo in a complex manner [35,36]. Finally, the detection of MVA-infected circulating hematopoietic cells within blood vessels corroborated that MVA is distributed via the blood stream to extraembryonic and embryonic tissues.

Apart from direct MVA infection of hepatocytes, the green color of the infected liver was another striking observation. The green color originated from canalicular and small ductular bile cholestasis and storage of bile pigment in Kupffer cells. Birds lack biliverdin reductase, thus the final product of avian heme catabolism and the predominant bile pigment is biliverdin [37]. Bile storage in Kupffer cells can be due to increased erythrophagocytosis and heme catabolism or regurgitation of bile pigments by hepatocytes. We ruled out obstructive cholestasis in the chicken embryos, because gall bladders and large bile ducts were normal and ductular reactions were not detected [38]. As histology and serum chemistry revealed at the most minor hepatocyte damage, cholestasis is likely not caused by hepatocyte damage but directly attributed to generalized infection. In mammals including humans, there is a so called “cholestasis of sepsis”, in which systemic pro-inflammatory cytokines like IL-1β and TNF-α lead to downregulation of bile acid transporter proteins and to canalicular and ductular cholestasis [39]. Typically, serum liver enzymes are not elevated and hepatocellular injury is absent. Increased breakdown of erythrocytes by intravascular or extravascular hemolysis can contribute to increased bile pigment production [40]. Viral infection of macrophages can lead to dramatically increased synthesis of pro-inflammatory cytokines in different species [41,42,43]. Interestingly, MVA-infected murine and human macrophage-like cells activate a robust chemokine production but a fairly weak inflammatory response. Hereby, bioactive IL-1β produced in response to MVA infection is effectively neutralized by the MVA encoded viral IL-1β-receptor protein [44,45,46]. Thus, it is tempting to speculate that the observed cholestasis is a consequence of a generalized inflammatory reaction possibly supported by a malfunction of orthopoxviral inhibitor protein(s) in the infected chicken embryos. Surprisingly, viral loads in the liver were reduced in embryonated eggs infected with 10^7^ IU of MVA and MVA-GFP-mCherry when compared with those from eggs infected with 10^3^ or 10^5^ IU of MVA. This observation might be correlated with an increased accumulation of bile constituents in the liver, which might interfere with in vivo or in vitro growth of MVA.

Overall, the inflammatory reaction to MVA infection predominantly consisted of the infiltration of granulocytes and macrophages and the expansion of granulopoiesis in liver and lung. This observation is somewhat untypical for a viral infection, which usually features lymphohistiocytic or lymphoplasmacytic infiltrates. Yet, inoculation of other orthopoxviruses on chicken embryo CAMs can result in a similar response. The white pock lesions in cowpox virus infections are largely comprised of granulocytes [47]. At the embryonic stage of our experimental infection (ed11), the embryonic acquired immune system is not fully functional, because colonization of lymphoid organs partially occurs at later time points, e.g., the thymus is colonized in three distinct waves on ed6.5, ed12, and ed18 [35]. In addition, chicken embryonic and early post-hatch T-cell responses are reduced when compared to those in chickens at later time points [48,49,50].

In 1975, Mayr and colleagues investigated the outcome of CAM inoculations with MVA or CVA. In comparison to CVA, the pock lesions of MVA on the CAM lacked typical morphology of necrosis, a significantly reduced rate of embryonic death was reported (40% compared to 100% with CVA) and embryonic skin lesions did not form upon MVA inoculation [2]. Also in our dose escalation study, we determined a clearly low virulence of MVA for chicken embryos. Even infections with the highest dose of 10^7^ IU MVA per embryo did not result in embryonic death. This is surprising, when considering that MVA does productively replicate and efficiently spread in the chicken embryos. Moreover, these chicken embryos are naturally immunocompromised during their ontogenesis, e.g., due to the lack of mature thymocyte responses [50]. Thus, these results support an exceptional safety profile of MVA and the MVA vaccine delivery system in chicken hosts as well.

Indeed, these findings are highly encouraging to further investigate the usefulness of recombinant MVA as vaccine vector system for immunization of birds and poultry including in ovo vaccination. In a first in ovo vaccination study in chicken using recombinant MVA, the authors did not observe any adverse effects, but distribution and pathogenesis of MVA infection were not examined [15]. The thorough investigation of MVA vaccine safety, immunogenicity and efficacy including the use of commercial egg injection machines and cost-efficient vaccination doses will clearly require further studies.

In summary, CAM inoculation represents an excellent model to study distribution and pathogenesis of different infectious agents, because the CAM is a highly vascularized organ, which functions as major site of gas exchange for the developing chicken. Main target cells of MVA infection in chicken embryos are Kul01+ macrophages, hematopoietic cells and hepatocytes. MVA doses up to 10^7^ IU do not lead to increased mortality, highlighting an excellent safety profile of this attenuated VACV. The chicken embryo infection may also be useful as a model to study virus–avian host interactions. In particular, it might be promising to reassess the function of poxviral proteins known to modulate innate responses of the mammalian immune system. Finally, first promising results from experimental MVA vaccination in poultry and the replication competence of MVA in the embryonated egg support future studies in vaccine development including in ovo vaccination.
